# A New Adenovirus Based Vaccine Vector Expressing an *Eimeria tenella* Derived TLR Agonist Improves Cellular Immune Responses to an Antigenic Target

**DOI:** 10.1371/journal.pone.0009579

**Published:** 2010-03-08

**Authors:** Daniel M. Appledorn, Yasser A. Aldhamen, William DePas, Sergey S. Seregin, Chyong-Jy J. Liu, Nathan Schuldt, Darin Quach, Dionisia Quiroga, Sarah Godbehere, Igor Zlatkin, Sungjin Kim, J. Justin McCormick, Andrea Amalfitano

**Affiliations:** 1 Department of Microbiology and Molecular Genetics, Michigan State University, East Lansing, Michigan, United States of America; 2 Department of Biochemistry and Molecular Biology, Michigan State University, East Lansing, Michigan, United States of America; The University of Chicago, United States of America

## Abstract

**Background:**

Adenoviral based vectors remain promising vaccine platforms for use against numerous pathogens, including HIV. Recent vaccine trials utilizing Adenovirus based vaccines expressing HIV antigens confirmed induction of cellular immune responses, but these responses failed to prevent HIV infections in vaccinees. This illustrates the need to develop vaccine formulations capable of generating more potent T-cell responses to HIV antigens, such as HIV-Gag, since robust immune responses to this antigen correlate with improved outcomes in long-term non-progressor HIV infected individuals.

**Methodology/Principal Findings:**

In this study we designed a novel vaccine strategy utilizing an Ad-based vector expressing a potent TLR agonist derived from *Eimeria tenella* as an adjuvant to improve immune responses from a [E1-]Ad-based HIV-Gag vaccine. Our results confirm that expression of rEA elicits significantly increased TLR mediated innate immune responses as measured by the influx of plasma cytokines and chemokines, and activation of innate immune responding cells. Furthermore, our data show that the quantity and quality of HIV-Gag specific CD8^+^ and CD8^−^ T-cell responses were significantly improved when coupled with rEA expression. These responses also correlated with a significantly increased number of HIV-Gag derived epitopes being recognized by host T cells. Finally, functional assays confirmed that rEA expression significantly improved antigen specific CTL responses, *in vivo*. Moreover, we show that these improved responses were dependent upon improved TLR pathway interactions.

**Conclusion/Significance:**

The data presented in this study illustrate the potential utility of Ad-based vectors expressing TLR agonists to improve clinical outcomes dependent upon induction of robust, antigen specific immune responses.

## Introduction

Although vaccines have provided the greatest impact on human health and disease, conventional vaccine technologies cannot elicit the robust immune responses required to eradicate some of the most pathogenic diseases to affect humanity, such as HIV-AIDS. Clearly, new paradigms that increase the potency of currently utilized vaccine platforms are required before diseases like HIV-AIDS can be treated or prevented. It is now proven that in humans, Adenovirus (Ad) based vaccines can induce potent cellular immune responses to HIV derived antigens (as compared to DNA or other virus based vaccine platforms) but that these responses do not reach the levels noted in so called long-term non-progressors [1], [2].

We, and others, have confirmed that the ability of Ad-based vaccines to induce innate and adaptive immune responses to Ad-expressed antigens is mediated by MyD88 and TRIF dependent intracellular signaling pathways [3], [4], [5], [6]. Since innate immune responses can dictate the quality of downstream adaptive immune responses, we have begun to explore the possibility of improving Ad-based vaccine efficacy by simultaneously enhancing toll-like receptor (TLR) pathway activation [7].

Relative to treatments with individual TLR ligands, exposure of sentinel cells of the innate immune system to combinations of TLR ligands results in synergistic inductions of pro-inflammatory factors such as IL-12p70, TNFα and type I IFNs (IFNα and IFNβ) [8]. This synergism can lead to increased activation of cytolytic cells, such as NK cells as but one example [9]. In addition to heightened innate immune responses, the addition of multiple innate receptor agonists in vaccine formulations can enhance the efficacy of pathogen or cancer cell specific humoral and cellular immune responses [10]. These findings suggested to us that combining Ad-based vaccine platforms with potent TLR ligands may result in a synergistic improvement in the induction of adaptive immune responses to Ad-vaccine expressed antigens.

One such potent TLR agonist is an *Eimeria tenella* expressed antigen, originally isolated based upon its ability to induce high levels of IL-12 in bovine intestine [11]. The gene encoding recombinant *E.tenella* antigen (rEA; NCBI Accession: AY745810), was first cloned from *E.tenella* oocyst mRNA [11], [12]. In a murine model, intra-peritoneal (i.p.) injection of purified rEA protein resulted in a dramatic induction of Th1 cytokines at 6 hours post injection (hpi) [11]. Additionally, *in vitro* studies showed that enhanced target cell killing was induced following treatment of NK-cell enriched splenocytes with rEA protein [11]. *In vivo*, purified rEA protein has illustrated the potential to increase protective immunity in animal models, and trigger innate immune responses in human clinical trial subjects [13], [14]. For these reasons, we hypothesized that expression of rEA from an Ad-based vector would generate significantly heightened innate responses leading to more robust adaptive immune responses against antigens expressed from the vaccine platform. In this study, we confirm that an Ad-based vector expressing rEA can generate stronger cellular immune responses to co-expressed or co-administered antigens than that generated by conventional Ad-based vaccines.

## Results

### Incorporation of rEA into an Ad-Vector Induces Enhanced Innate Immune Responses *In Vivo*

**Figure 1:**
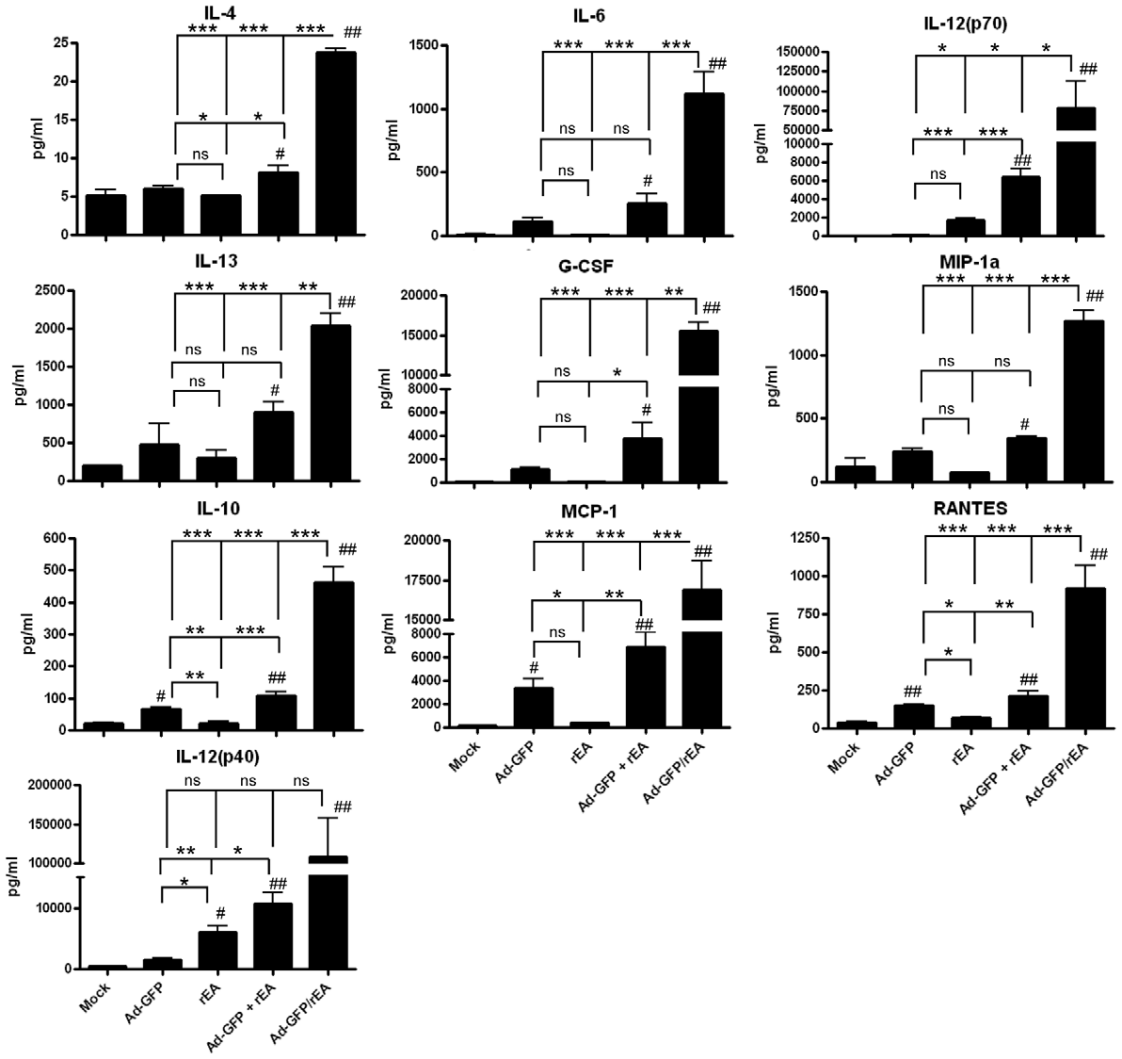
Heightened Ad-GFP/rEA induced cytokine responses. (**A**) C57BL/6 mice were mock injected (n = 4), or IV injected with 7.5×10^10^ vps of Ad-GFP, 1 ng purified rEA protein, a mix of Ad-GFP and rEA protein, or Ad-GFP/rEA. Plasma was harvested and cytokines and chemokines were measured at 6 hpi (n = 5-10). Bars represent mean ± SE. #, ## represent significantly different from mock injected animals (p<0.05, p<0.01 respectively). *,**, *** represent statistical differences (p<0.05, p<0.01, p<0.001, respectively). ns, not significant.

We first sought to determine if co-injection of both the TLR agonist protein rEA with an Ad-based vector would synergistically induce heightened cytokine and chemokine responses as compared to either treatment alone following intravenous administration, a common strategy used to characterize Ad-induced innate immune responses *in vivo*. To accomplish this, we co-administered 1 ng of purified rEA protein with 7.5×10^10^ vps of Ad-GFP, and compared the ensuing cytokine and chemokine responses to those generated in mice injected with either Ad-GFP or rEA alone (Figure 1). At 6 hpi, serum levels of IL-4, IL-6, IL-12(p70), IL-13, G-CSF, and MIP-1a were significantly induced over background levels only in mice injected with both Ad-GFP and purified rEA protein (p<0.05). Furthermore, serum levels of IL-10, MCP-1, RANTES and IL-12(p40) were significantly higher in mice injected with both rEA and Ad-GFP when compared to those administered either treatment alone (p<0.05).

To determine if rEA *expressed* from an Ad vector might synergistically provoke a similar response, plasma cytokine and chemokine levels were measured following intravenous injection of a novel rEA expressing vector, Ad-GFP/rEA (Figure 1; Figure S1 *A*). Interestingly, at 6 hpi, we found that all plasma cytokines and chemokines tested were induced to higher levels with Ad-GFP/rEA than with either treatment alone or in combination (p<0.05) with the exception of KC and IL-12(p40); although a similar trend was still observed in these latter cytokines.

It has recently been shown that the release of both IL-12 and IFNγ following rEA treatment requires the TLR adaptor protein MyD88 [15]. To evaluate the potential role for TLR signaling in mediating the enhanced innate immune responses observed after Ad-GFP/rEA injection, we also completed an identical experiment in mice genetically deficient in MyD88. The resulting data show that only slight inductions of IL-12 at 1 and 6 hpi, MCP-1 at 1 hpi, and RANTES at 6 hpi, occurred in the Ad-GFP/rEA injected MyD88-KO mice, elevations that were insignificant relative to the robust rEA induced responses we noted in similarly treated wild-type C57BL/6 mice (Figure S1 *B*). These data confirm that MyD88 mediated TLR signaling is at least partially, if not completely, required for the robust innate immune responses noted shortly after injection of Ad-GFP/rEA.

The rapid induction of these innate factors correlated with the transcription of the Ad transduced rEA gene within 6 hours of vector administration (Figure S2). It is noteworthy that we also detected higher levels of GFP encoding RNA as well as GFP protein in both the liver and the spleen of Ad-GFP/rEA injected mice as compared to control mice. In contrast, similarly heightened levels of GFP specific RNA transcripts or protein were not observed in MyD88-KO mice identically injected with the Ad-GFP/rEA virus. Known NFκB binding sites within the CMV promoter/enhancer region may explain this result; i.e. rEA induced, MyD88 dependent induction of NFκB activity may result in increased transcription of CMV driven transgenes [16]. Importantly, the robust innate immune response elicited by Ad-GFP/rEA was attenuated when the virus was UV-inactivated (rendering GFP and rEA expression nearly undetectable) prior to injection (Figure S2, S3). This indicates that *de novo* expression of rEA was primarily responsible for the observed results.

Other measures of Ad induced innate immune responses revealed that thrombocytopenia was significantly greater at both 1 and 3 dpi (p<0.01) in mice treated with Ad-GFP/rEA as compared to control mice (Figure S4 *A*). However, the kinetics of systemic ALT elevations were not significantly different between the Ad-GFP and the Ad-GFP/rEA treated mice (Figure S4 *B*).

### Ad-Vectors Expressing rEA Induce Enhanced Innate Immune Cell Activation

**Figure 2:**
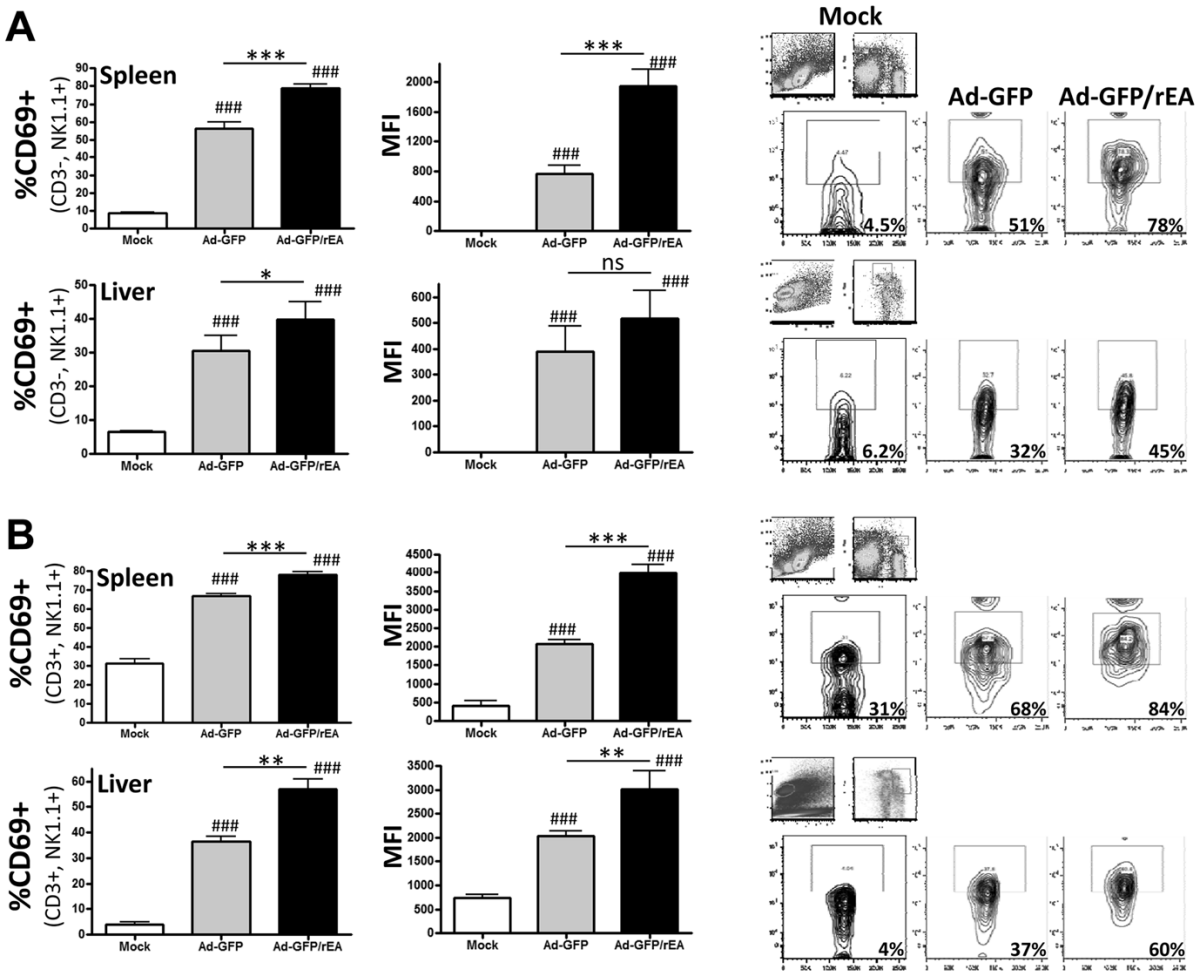
Ad-GFP and Ad-GFP/rEA induced NK and NKT cell activation in liver and spleen *in vivo*. C57BL/6 mice (N = 3) were either mock injected or injected with 7.5×10^10^ vps of either Ad-GFP or Ad-GFP/rEA. Lymphocytes from liver and spleen tissue were harvested at 6 hpi, stained for expression of surface markers for (A) NK cells or (B) NKT cells and FACS sorted. Bars represent mean ± SE. #, ##, ### represent significantly different from mock injected mice (p<0.05, p<0.01 respectively). *,**,*** represent statistical differences (p<0.05, p<0.01, p<0.001, respectively). Representative contour plots are illustrated at right. ns, not significant; MFI, mean fluorescence intensity.

The activation of NK cells has been shown to occur subsequent to Ad administrations, as well as recombinant rEA protein injections [11], [17]. Our results confirm that at 6 hpi, both Ad-based vectors and purified rEA protein individually induced a rapid activation of splenic NK and NKT cells as measured by the increase in percentage of these cell types that express the lymphocyte activation marker CD69 (data not shown). However co-administration of both the Ad-vector and purified rEA protein did not result in a synergistic induction of CD69 on the surface of either NK or NKT cells derived from the spleen (data not shown). More impressively, our data show that expression of rEA from the Ad-GFP/rEA vector not only resulted in a significant increase in the percentage of CD69 positive NK and NKT cells present in both the liver and the spleen (p<0.05) (Figure 2*A* and *B*), but also indicated that the per cell expression of CD69 (as measured by the mean fluorescence intensity (MFI)) is also significantly higher on the surface of both NK and NKT cells in the spleen, as well as NKT cells in the liver (p<0.01). After Ad-GFP control vector treatment, we observed a significant decrease in the total population of NK cells in the spleen that coincided with a significant increase of NK cells in the liver while the total NKT cell population decreased in both the liver and the spleen (Figure S5 *A*). No significant differences in NK/NKT cell distribution were observed between Ad-GFP and Ad-GFP/rEA injected mice.

**Figure 3:**
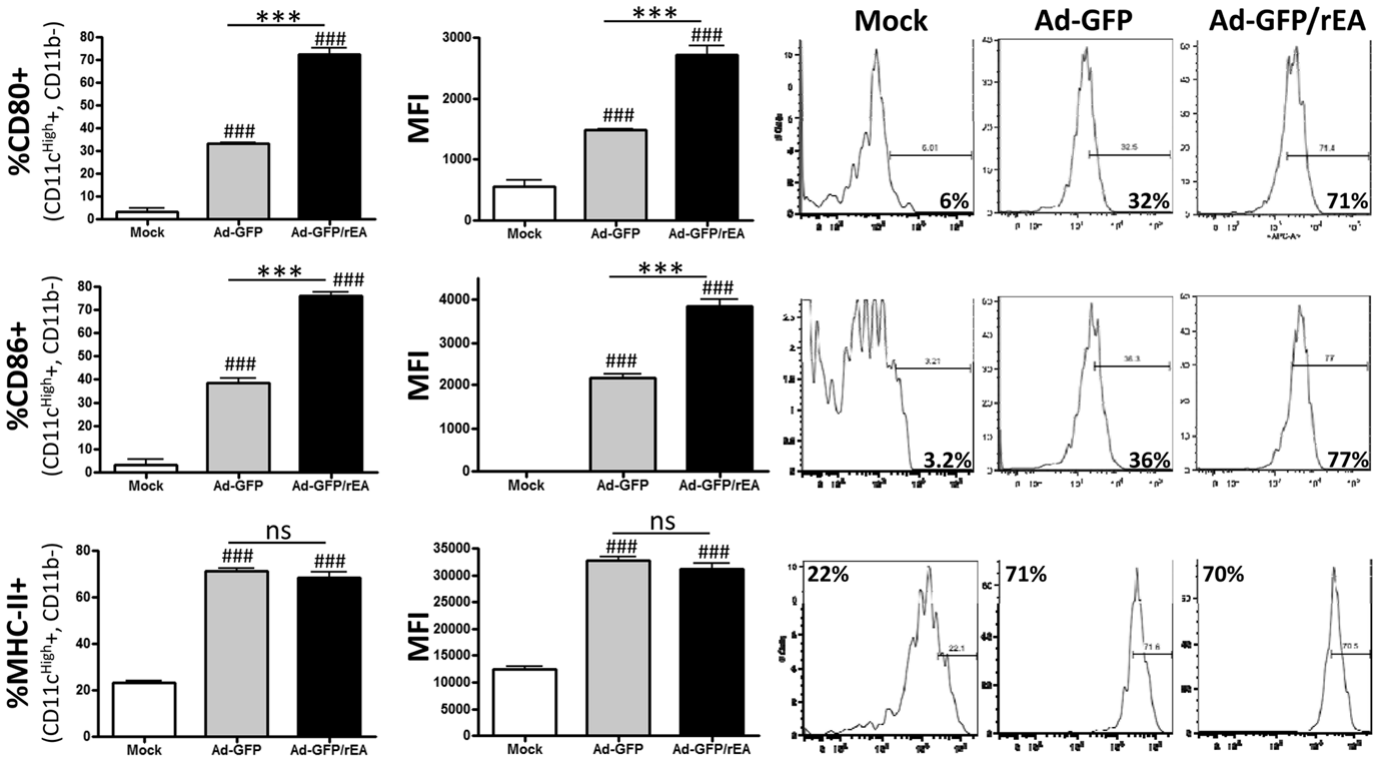
Ad-GFP and Ad-GFP/rEA induced DC activation in spleen *in vivo*. C57BL/6 mice (N = 3) were either mock injected or injected with 7.5×10^10^ vps of either Ad-GFP or Ad-GFP/rEA. Lymphocytes from spleen tissue were harvested at 6 hpi, stained for expression of surface markers Cd11c, Cd11b, CD80, CD86 and MHC-II. CD-19, NK1.1, and CD3 expressing cells were eliminated using PerCP-Cy5.5 labeled antibodies. Cd11c^High^, Cd11b- cells are represented. Bars represent mean ± SE. #, ##, ### represent significantly different from mock injected mice (p<0.05, p<0.01, p<0.001 respectively). *,**,*** represent statistical differences (p<0.05, p<0.01, p<0.001, respectively). Representative histograms are found at right. ns, not significant; MFI, mean fluorescence intensity.

It has been shown that administration of rEA protein can induce heightened DC activation and maturation *in vitro* which results in the release of both IFNγ and IL-12 [18]. Furthermore, numerous TLR agonists have been shown to increase the expression of MHC class II molecules on antigen presenting cells (APCs) [19]. Although we found a statistical increase in the percentage of MHC-II expressing Cd11b+ (a macrophage marker) splenocytes in mice injected with both Ad-GFP and rEA protein as compared to mock injected mice, no statistical differences in MHC-II expressing Cd11b+ or Cd11c+ (DC marker) splenocytes were observed in mice co-injected with rEA and Ad-GFP versus those mice injected with either treatment individually (data not shown). Furthermore, we did not detect significantly higher percentages of MHC-II+ macrophages or DCs in mice injected with Ad-GFP/rEA as compared to those injected with Ad-GFP (Figure 3 and data not shown).

Both CD80 and CD86 are considered important co-stimulatory molecules present on DCs that enhance the activation of both B-cells and T-cells. Therefore we also evaluated the induction of both of these factors on the surface of DCs (Cd11c^High^, Cd11b-) derived from the spleens of Ad-GFP or Ad-GFP/rEA injected mice. Our results confirm that both CD80 and CD86 co-stimulatory molecules are induced by injection of Ad-based vectors alone, and that expression of rEA from Ad-GFP/rEA results in a significant increase in the percentages of splenic DCs expressing these factors when compared to mice identically treated with Ad-GFP (p<0.001) (Figure 3). Furthermore MFI analyses indicate that the per cell expression of both CD80 and CD86 is also significantly higher on the surface of CD11c+, Cd11b- splenocytes derived from Ad-GFP/rEA injected mice (p<0.001). We also observed a dramatic shift in the Cd11b/Cd11c expressing splenocyte population following Ad-vector injections. Specifically, whereas the population of Cd11c+ cells significantly increased (from 5% to >35%), the percentage of splenocytes expressing Cd11b significantly decreased (from 15% to <5%) likely due to the migration and/or destruction of Ad infected Cd11b+ and Cd11c+ APCs in the spleen (Figure S5 *B*).

### An rEA Expressing Ad Vector Can Generate Improved T-Cell Responses to Co-Expressed Antigenic Targets

To determine if purified rEA protein mixed with the Ad-Gag vaccine could serve as an adjuvant to induce improvements in Gag specific cellular mediated immune (CMI) responses, C57BL/6 and Balb/c mice were injected IM with Ad-Gag ± various doses of purified rEA protein (1, 10, or 100 ng) (Figure S6). At 14 dpi, splenocytes were isolated from vaccinated animals and exposed to known immunodominant Gag peptides to recall a memory response *ex vivo*. We did not observe improved CMI responses in mice treated with the rEA protein adjuvanted vaccine at any dose tested (Figure S6). In fact, we detected significantly lower Gag specific CMI responses in Balb/c mice injected with Ad-GFP +100 ng purified rEA. The reason for such an unexpected effect is unclear, but has been observed in other model systems in which a TLR9 CpG agonist was used in combination with an Ad-based vaccine formulation [20]. A comparison of the plasma cytokine and chemokine profile in mice injected IM or IP with purified rEA protein did not reveal significant differences, thus eliminating the possibility that route of rEA adjuvant injection could partially explain this response (data not shown).

**Figure 4:**
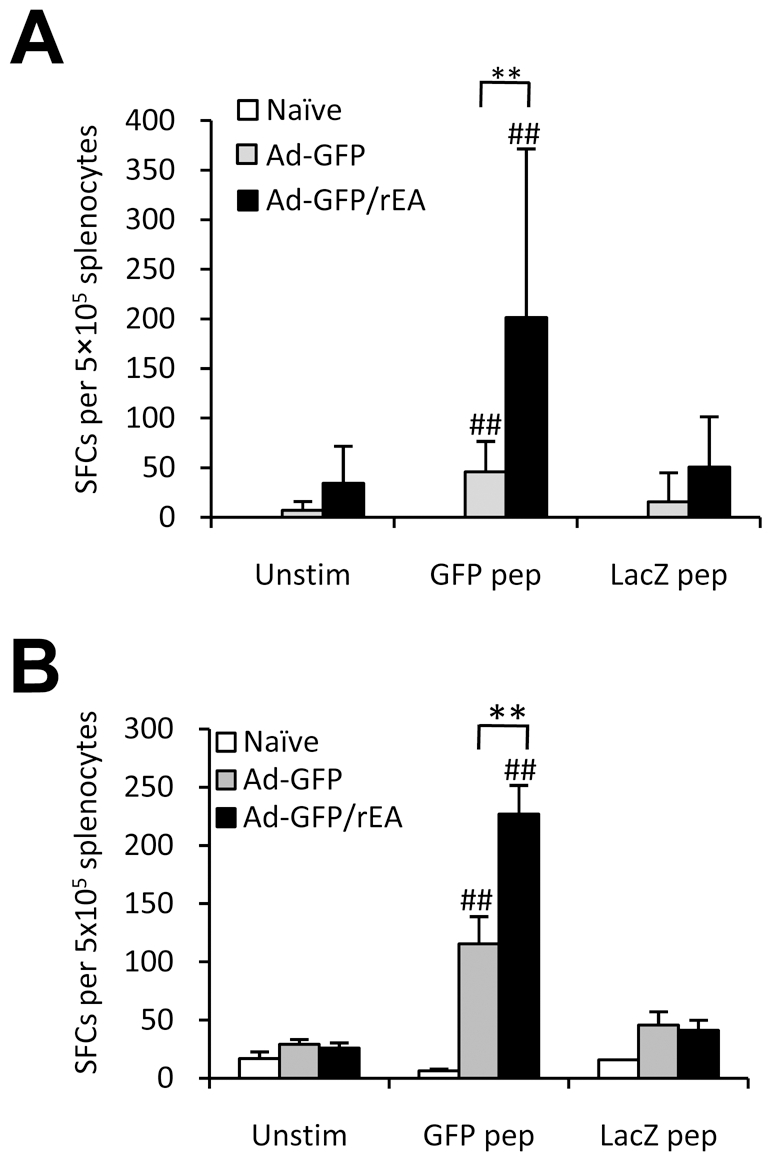
Heightened transgene specific cellular responses as a result of Ad-GFP/rEA injection. (**A**) C57BL/6 mice were mock injected (n = 3) or IV injected with 1×10^10^ vp of either Ad-GFP or Ad-GFP/rEA (n = 8). Splenocytes were harvested 7 dpi and used to complete IFNγ ELISpot analyses. Spot forming cells (SFCs) are reported. (**B**) C57BL/6 mice (n = 6) were injected IM as above. IFNγ ELISpot analyses were completed at 14 dpi. Bars represent mean ± SE. ## represent significantly different from mock injected mice (p<0.01). *,** represent statistical differences (p<0.01).

We next evaluated antigen specific CMI responses generated against transgenes expressed by conventional, or rEA co-expressing Ad-vectors. At 7 dpi, mice intravenously treated with the Ad-GFP/rEA virus demonstrated a significantly higher GFP specific CMI response when compared to control animals as measured by IFNγ ELISpot analyses (p<0.01) (Figure 4*A*). Similar results were obtained after intramuscular vaccinations (p<0.01) (Figure 4*B*). Importantly, these results indicate that an Adenovirus vector *expressing* a TLR agonist such as rEA has a superior ability to enhance antigen specific CMI responses *in vivo* relative to co-administration of purified TLR agonist proteins.

**Figure 5:**
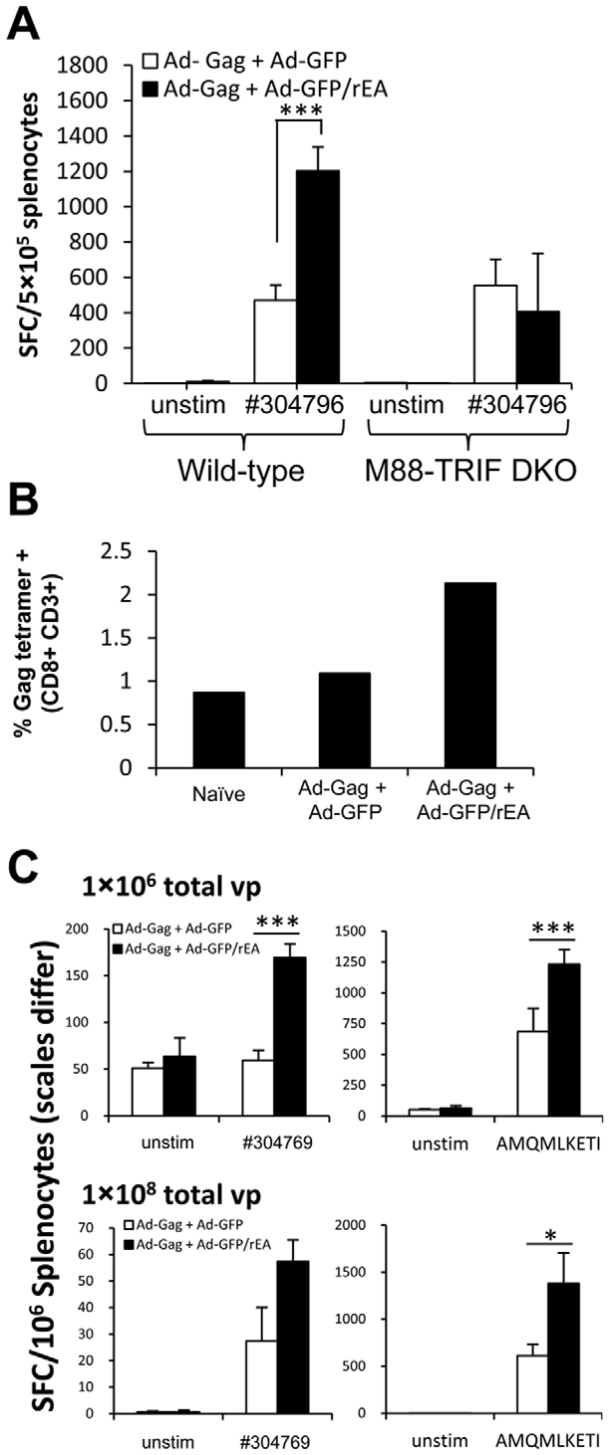
Increased HIV-Gag specific CMI responses in mice co-injected with Ad-GFP/rEA. (**A**) Wild-type (N = 3) or MyD88/TRIF double knockout (M88-TRIF DKO; N = 3) mice were co-immunized IM with equivalent viral particles of Ad-Gag mixed with either Ad-GFP or Ad-GFP/rEA (total of 1×10^7^ vps mixed prior to injection). At 14 dpi IFNγ ELISpot analyses were completed using QBI# 304796 to restimulate cells *ex vivo*. Data are presented as mean ± SE. *** denotes statistically significant difference (p<0.001). (**B, C**) Balb/c mice (n = 3) were co-immunized IM as above (total of 1×10^6^ vps). At 8 dpi, Gag specific CD3+/CD8+ T-cells in PBMC preparations were analyzed by FACS. (**C**) At 14 dpi, splenocytes were stimulated *ex vivo* with the indicated peptide to complete IFNγ ELISpot analyses. Data represent the mean ± SE. *, *** represent p<0.05, p<0.001 respectively. This experiment was completed in triplicate at various doses yielding identical results (two doses are illustrated in this figure).

To determine if an Ad expressing rEA could facilitate improved adaptive immune responses to pathogen specific antigens, we evaluated the induction HIV-Gag antigen specific CMI responses in splenocytes harvested from C57BL/6 mice co-immunized with equivalent particle numbers of an Ad-based vaccine expressing the HIV-1 clade B Gag protein mixed with either Ad-GFP, or Ad-GFP/rEA. At 14 dpi, we observed a >100% improvement in HIV-Gag specific CMI responses when Ad-GFP/rEA was present (p<0.001) (Figure 5*A*). Importantly, this improved response was not observed in MyD88/TRIF double knockout mice confirming the requirement for MyD88/TRIF dependent TLR pathways in mediating this response (Figure 5*A*).

**Figure 6:**
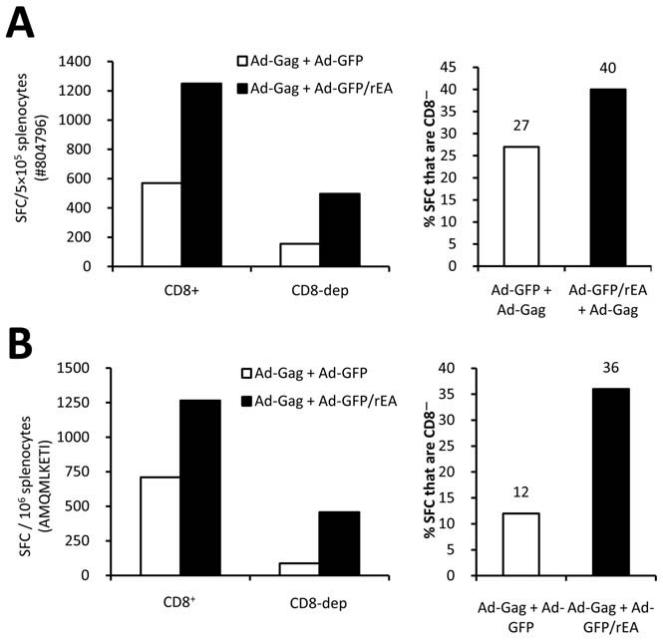
Ad-GFP/rEA improves antigen specific CD8^+^ and CD8^-^ cellular responses. At 14 dpi, splenocytes from vaccinated C57BL/6 (1×10^7^ total vps) (**A**) or Balb/c (1×10^8^ total vps) (**B**) mice were equivalently pooled (N = 3 mice per treatment) and CD8+ cells were depleted using magnetic beads and IFNγ ELISpot analyses were completed. %SFC that are CD8^-^ = (#SFCs CD8 dep/#SFCs CD8^+^)*100. This experiment was repeated two independent times with the same result.

To determine if rEA expression facilitates adaptive immune responses despite differences in genetic backgrounds, we performed similar experiments in Balb/c mice. At 8 dpi, we detected significantly more Gag specific CD8^+^ T-cells in PBMC preparations 8 days post vaccination in mice co-injected with the Ad-Gag and Ad-GFP/rEA vectors using a Gag specific MHC class I tetramer (Figure 5*B*). Furthermore, significantly heightened HIV-Gag specific IFNγ expressing T-cells were detected in Ad-GFP/rEA treated mice at 14 dpi when splenocytes were stimulated *ex vivo* with two different HIV-Gag derived peptides (Figure 5*C*). Following CD8^+^ T-cell depletion, our results indicated that only 10-30% of the HIV-Gag specific, IFNγ secreting cells were CD8^−^ in both C57BL/6 (Figure 6*A*) and Balb/c (Figure 6*B*) mice immunized with Ad-Gag+Ad-GFP. Interestingly, this ratio was altered in mice co-vaccinated with Ad-GFP/rEA, where rEA expression facilitated a more balanced response, manifesting as an increased generation of greater numbers of CD8^−^ cells (most likely CD4^+^ T-cells, and/or a very minor population of NK and/or NKT cells) in the antigen specific responsive T-cell population.

### Ad Vaccines Expressing TLR Agonists Increase the Breadth of HIV-Gag Specific CMI Responses

**Figure 7:**
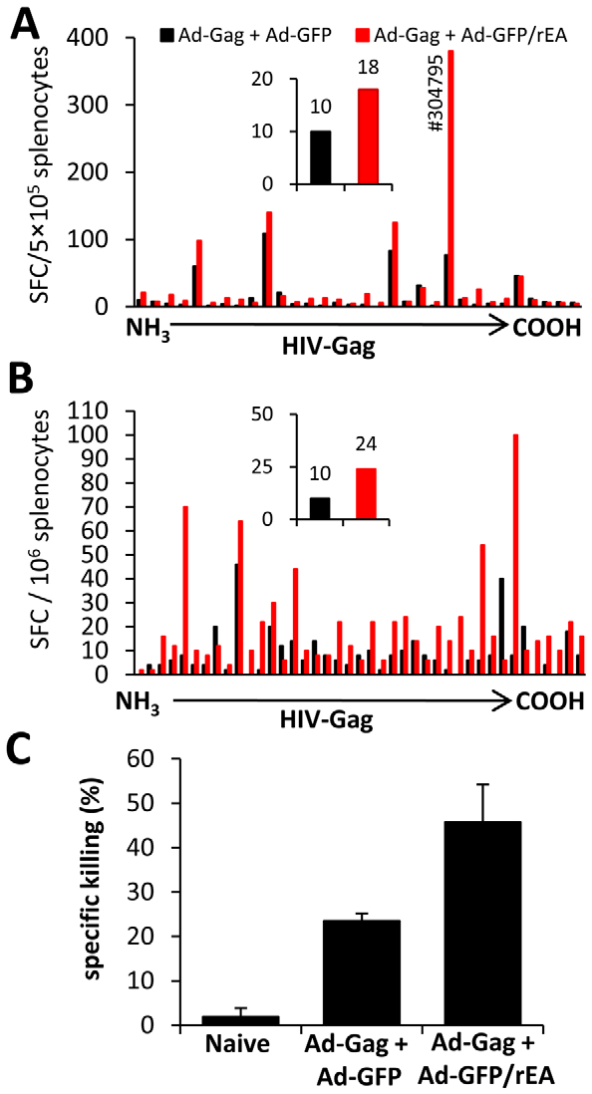
Increased quantity and quality of Gag-specific T-cells in Ad-GFP/rEA co-injected mice. At 14 dpi, splenocytes from vaccinated C57BL/6 (1×10^7^ total vps) (**A**) or Balb/c (1×10^8^ total vps) (**B**) mice were equivalently pooled (N = 3 mice per treatment) and individual wells were stimulated *ex vivo* with a pool of 2-4 15-mer peptides spanning the complete Gag protein sequence, not including peptides included in Figure 5 (AMQ, or 304796). Inset graphs indicate the number of wells with >10 SFCs. This experiment was carried out two independent times with the same result. (C) Balb/c mice were vaccinated as above (total of 1×10^7^ vp). At 14 dpi, an *in vivo* CTL assay was completed and Gag specific CTL activity was quantified (N = 2 for all treatments). This experiment was carried out two independent times, each with the same result.

Increased breadth of CMI responses has been associated with improved protection upon pathogenic challenge [21]. To measure the breadth of the HIV-Gag specific CMI response, we harvested splenocytes from both C57BL/6 (Figure 7*A*) and Balb/c (Figure 7*B*) mice co-injected with the Ad-Gag and Ad-GFP/rEA vectors, and stimulated the cells *ex vivo* with peptide pools each containing 2-4 HIV-Gag specific overlapping 15mer peptides spanning the entire HIV-Gag protein sequence. We observed a dramatic increase in the breadth of the HIV-Gag specific CMI response induced by utilization of the rEA expressing Ad, as evidenced by greater numbers of peptide pools activating splenocytes from Ad-GFP/rEA treated mice. These data indicate that expression of rEA facilitated the recruitment of greater numbers of diverse HIV-Gag specific T-cell clones, each reactive to unique HIV-Gag specific peptides present within the entire HIV-Gag protein sequence.

To determine if this improved induction of T-cell responses translated into improvements in antigen specific T-cell effector functions, we confirmed that Balb/c mice co-vaccinated with the Ad-GFP/rEA and Ad-Gag vectors have a >80% improvement in HIV-Gag specific cytotoxic T-cell mediated killing *in vivo* (Figure 7*C*). These data indicate that expression of rEA not only increases the quality and breadth of several antigen specific CMI responses, but also facilitates an increased functional capacity of antigen specific T-cells to kill cells presenting a pathogen specific peptide, in this instance an antigen derived from the HIV-Gag protein.

## Discussion

Our work, and that of others, suggest that TLR mediated signaling may be an important reason as to why Ad based vaccines appear to be superior to other vaccine platforms, especially in regard to elicitation of antigen specific cellular (T-cell) immune responses [3], [5], [6], [22]. Building upon these findings, we hypothesized that synergistic manipulation of TLR dependent innate immune responses may further improve the ability of Ad-based vaccines to induce beneficial immune responses to pathogen derived antigens. The mechanism by which TLR agonists function as adjuvants is not completely understood, however it is presumed that enhanced release of co-stimulatory factors (such as pro-inflammatory cytokines) from antigen presenting cells improves CD8^+^ T cell priming, possibly via facilitation of more efficient antigen processing and MHC class I and II antigen presentation. The data presented in this study illustrate that *de novo* expression of a TLR agonist, such as rEA, from an Ad-based vector can dramatically increase the release of co-stimulatory cytokines and chemokines, the majority of which were TLR system dependent. These inductions correlated with an increase in DC activation in the spleen, as well as NK and NKT cell activation in both the liver and the spleen. The increased activation of these cell types immediately following Ad-GFP/rEA vaccination likely contributed to both the increase in population and functionality of antigen specific cells noted in this study [23], [24]. We also observed enhanced thrombocytopenia following Ad-GFP/rEA administration, an innate immune response that positively correlated with other innate immune responses evaluated after administration of the rEA expressing Ad vector. Importantly, we did not observe an increase in liver toxicity after IV administration of the rEA expressing Ad5 vaccine, a result that is in stark contrast to results we have previously reported following injection of other human and non-human Ad serotypes currently being considered as alternative Ad-based vaccine platforms [25].

Most recently, the STEP trial revealed that use of a conventional Ad5 based vaccine expressing HIV antigens induced significant HIV specific cellular immune responses, yet the vaccine failed in preventing HIV infections in vaccinees [26]. Although the results of the STEP trial were confounded by a low acquisition rate of HIV by Ad5 immune, placebo treated vaccinees, the data resulting from this trial could suggest that uncircumcised males with pre-existing Ad5 immunity may have contracted the HIV virus at a greater rate than placebo control populations. Ongoing investigations into the mechanisms behind these latter notions have been contradictory and include conflicting studies as to whether or not HIV transmission is also a result of robust CD4+ T-cell responses to the Ad5 vector in Ad5 immune vaccinees [27], [28]. Newly initiated studies utilizing Ad based HIV vaccines in Ad-naive vaccinees should help clarify these issues.

Recent evidence has suggested that a robust CTL proliferative response to the HIV-Gag antigen in long term non-progressors infected with HIV is the most common immune parameter associated with controlling viral loads [1]. It is therefore possible that the disappointing outcome in the STEP trial may be correlative to the conventional Ad vaccine's sub-optimal elicitation of HIV-Gag specific T cell responses in vaccinees [26]. Therefore, we sought to determine if use of an Ad5 vaccine expressing a TLR agonist could improve immune responses to the HIV-Gag antigen. The TLR agonist rEA has been shown in mouse and human clinical trials to correlate with functional improvements in the quality of the innate and/or adaptive immune responses elicited [11], [13], [14]. Our experiments confirmed a heightened HIV-Gag specific T-cell response in PBMC preparations, and a significant improvement in the numbers, and antigenic breadth of HIV-Gag specific, IFNγ expressing T-cells present in splenocytes derived from animals treated with both HIV-Gag and TLR agonist (rEA) expressing Ad vaccines. Most importantly, the qualitative improvements elicited by Ad mediated expression of rEA positively correlated with an improved functional capability (CTL activity) of HIV-Gag specific T-cells *in vivo*. T-cell depletion studies confirmed that Ad mediated expression of rEA significantly altered the antigen specific CD4^+^/CD8^+^ T cell ratio, fostering induction of a more balanced number of the two antigen specific T-cell subsets in Ad-GFP/rEA treated animals.

Together, these data indicate that simultaneous expression of rEA, or potentially other similar TLR ligands from an Ad vector, can serve to enhance CMI responses to pathogen derived antigens expressed from either the same, or other Ad-based vectors. Although we have focused on Ad-based vaccine approaches, this platform could be expanded to other viral and non-viral vaccine formulations, and may also be useful in other immunization protocols including those employing whole organism or subunit vaccines. Of particular importance, these results confirmed the efficacy of this regimen in two independent mouse backgrounds that induce highly divergent immune responses in response to antigen exposure (i.e.: C57BL/6 mice initiate Th1 responses, while Balb/c mice initiate Th2 responses), which could be confirmed using indicator, as well as pathogen derived (e.g.: HIV-Gag) antigens. Together, these data suggest that this novel platform has the potential to succeed in multiple genetic backgrounds and against other diseases amenable to vaccine therapy. Future studies in higher order animal challenge models will be required to evaluate the potential for Ads expressing TLR agonists to be used as improved vaccine vectors. However, the facts presented in this study suggest that utilization of Ad-based vaccines expressing TLR agonists may show significantly improved efficacy in human populations.

## Methods

### Vector Construction

The Open Reading Frame (ORF) of rEA gene was inserted into an identical CMV driven cassette directly upstream of the GFP cassette. The resulting pAdTrack-rEA shuttle with the pAdEasyI Ad5 vector genome as previously described [29] yielding pAd-GFP/rEA. HEK293 cells were used to amplify virus. Direct sequencing and restriction enzyme mapping were carried out to confirm the integrity of the rEA sequence. A direct comparison of transduction efficiency was completed for Ad-GFP and Ad-GFP/rEA virus preparations using flow cytometry. Similar transduction efficiencies were observed (Figure S7). To construct Ad-Gag, the HXB2 Gag gene (Genbank Accession #K03455; kindly supplied by Dr. Frank Jones, Etubics, Corp., originally obtained from NIH Vaccine Research Center) was sub-cloned from the pvrc3900 plasmid into the pShuttle-CMV plasmid using restriction endonuclease cloning strategies. Restriction digests and sequencing were used to confirm the sequence integrity of the resulting shuttle (pShuttle-CMV-HIV-Gag). Recombination and viral propagation was completed as described previously [30]. Gag expression was verified using Western blotting (data not shown).

### Animal Procedures

All animal procedures were approved by the Michigan State University Animal Care and Use Committee. Intravenous injection of animals (2–4 months in age) via the retro-orbital sinus consisted of virus diluted in 200 µl of PBS (pH 7.4). Intramuscular injections were completed in a total volume of 20 µl into the tibialis anterior of the right hindlimb. Adult male C57BL/6 and Balb/c mice were purchased from The Jackson Laboratory (Bar Harbor, ME). MyD88-KO, and TRIF-KO mice were kindly provided by Dr. Shizuo Akira. MyD88/TRIF DKO mice were bred at Michigan State University. Plasma and tissue samples were processed at the indicated times post-injection as previously described [3].

### rEA Protein Purification

rEA protein was prepared as previously described with minor modifications [11]. rEA protein from *E. tenella* was expressed from pET Blue1 plasmid in Rosetta2 DE3 pLacI strain of *E.coli* as native non-tagged protein. Bacterial growth and IPTG induction of the gene were optimized according to manufacturer's recommendations. Bacterial cells were collected, washed twice with phosphate-buffered saline (PBS), re-suspended in PBS supplemented with 4 mM PMSF and disrupted with 5 mg/ml lysozyme (Worthington, Freehold, NJ) and sonication (Sonifier Cell Disruptor 350, Branson, Danbury, CT) (15 pulses, 10 sec each with 1 min intervals on ice) followed by high-speed centrifugation to remove cell debris. The protein was then purified from bacterial lysates by a modification of the procedure described previously [11].

#### 1. Ammonium sulfate (AS) fractionation

Bacterial lysate was fractionated with AS at 40% (1^st^ step, rEA in supernatant) and 80% (2^nd^ step, rEA in pellet) saturation. Final pellet was collected by high-speed centrifugation.

#### 2. DEAE chromatography

Protein sample was loaded onto DEAE-Sepharose B in 0.2M NaCl in PBS and eluted in 0.5M NaCl in PBS.

#### 3. Digestion of nucleic acids

rEA-containing fractions were treated with a mixture of protease-free RNase A (Boehringer, Columbus, OH) and DNase I (Roche, Indianapolis, IN), 4 hours at +4°C, 0.2 µg/ml, and 2 U/ml as final concentrations, respectively.

#### 4. Re-chromatography on DEAE-Sepharose B as in step 2

#### 5. Endotoxin removal

The sample was passed through Endotoxin Removing Detoxi Gel (Pierce, Rockford, IL) 3-4 times. Removal of endotoxin was followed by two tests: Pyrogent Plus test (Cambrex Bio Sci., Walkersville, MD) and Pyrochrome test (Associates of Cape Cod Inc., Falmouth, MA).

rEA protein in final preparation was found to be >95% homogeneous by SDS-gel electrophoresis and its concentration was determined by Bradford reagent (Bio-Rad). For mouse injections the final rEA preparation was diluted in 0.1% human serum albumin (HSA), in 0.9% saline (USP grade, Abbott Labs, Abbott Park, IL) and filter-sterilized.

### Cytokine and Chemokine Analysis

A mouse multiplex based assay was used to determine the indicated cytokine/chemokine plasma concentrations per the manufacturer's instructions (Bio-Rad, Hercules, CA) via Luminex 100 technology (Luminex, Austin, TX) essentially as previously described [3].

### Isolation of Lymphocytes

Splenocytes were prepared by physical disruption of spleen tissue, passage of the resulting cell suspension through a 40 µm sieve, followed by RBC lysis and resuspension in RPMI 1640 supplemented with 10% FBS and penicillin/streptomycin/fungizone. Liver tissue was minced into small pieces, incubated for 1 hour at 37°C with vortexing at 15 min intervals in collagenase/DNase containing RPMI 1640 supplemented with 10% FBS and penicillin/streptomycin/fungizone. This was followed by passing the tissue through a 40 µm sieve followed by RBC lysis and resuspension in complete RPMI media.

### ELISpot Analysis

ELISpot assays were completed per manufacturer's protocol using the Ready-set Go IFNγ mouse ELISpot kit purchased from eBioscience (San Diego, CA). 4 µg/ml of GFP, LacZ, or Gag specific peptides (GFP-DTLVNRIEL; LacZ–DAPIYTNV; Gag-AMQMLKETI) (Genscript, Piscataway, NJ) were used to stimulate 5×10^5^ splenocytes/well *ex vivo*. 15mer Gag specific peptides were obtained from the NIH AIDS Reagent and Reference Program Cat# 8117 Lot# 9. Spots were counted using an automated ELISpot reader system (Cellular Technology, Cleveland, OH).

### Cell Staining and Flow Cytometry

Splenocytes were stained with various combinations of the following antibodies: PE-CD69 (3 µg/ml), PE-Cy7-NK1.1, APC-CD3 (for NK and NKT cell activation), and PE-Cy7-CD11c, APC-Cy7-CD11b, APC-CD80, V450-CD86, Alexa Fluor700-MHC-II, PerCpCy5.5-CD19, PerCpCy5.5-CD3, and PerCpCy5.5-NK1.1 (for APC activation/maturation) (4 µg/ml) (All obtained from BD Biosciences, San Diego, CA). Cells were initially incubated with CD16/32 FcR II/III antibody to minimize nonspecific binding. Cells were then incubated on ice with the appropriate antibodies for 30 minutes, washed, and sorted using an LSR II instrument and analyzed using FlowJo software. For APC analysis, NK-, B-, and T cells were removed from the analysis using PerCpCy5.5-conjugated CD3, CD19, and NK1.1 antibodies. For tetramer staining, PBMCs were stained with PE labeled AMQMLKETI tetramer (NIH Tetramer Core Facility), APC-CD3, and Pacific Blue-CD8a. All antibodies were purchased from BD Biosciences, San Diego, CA. CD8^+^ cells were depleted from pooled splenocyte preparations using MACS beads per the manufacturer's protocol (Miltenyi Biotec, Bergisch Gladbach, Germany). FACS analyses revealed >96% depletion of CD8^+^ T-cells (Figure S8). All FACS studies were completed using an LSR II instrument and analyzed using FlowJo software.

### *In Vivo* CTL Assay

14 days post vaccination of Balb/c mice, syngeneic splenocytes from Naïve mice were pulsed with an irrelevant peptide (NYDNAGTNL) or with the HIV-Gag AMQMLKETI peptide and stained with 1 µM CFSE (CFSE^Low^) or 10 µM CFSE (CFSE^High^), respectively. Recipient animals were intravenously injected with equivalent amounts of both CFSE^Low^ and CFSE^High^ stained cells. Mice were terminally sacrificed 5 hpi, splenocytes were recovered, and sorted on an LSRII flow cytometer. FlowJo software was used to determine percentages of CFSE stained cells. % specific killing = 1-((% CFSE^High^/% CFSE^Low^)_immunized_/(% CFSE^High^/% CFSE^Low^)_non-immunized_).

### Supporting Methods

Additional methods are detailed in Methods S1 which describe the procedures used to complete the supporting experiments.

### Statistical Analysis

Statistically significant differences in innate factors were completed as follows: 1) ANOVA with a Dunnet's post hoc test was applied to determine significant inductions of factors relative to baseline (mock) values, 2) ANOVA with Neuman-Keuls post hoc test was applied to Ad-GFP, rEA, and Ad-GFP+rEA groups to determine if co-administration of both factors resulted in significantly higher levels of each factor, and 3) ANOVA with Neuman-Keuls post hoc test was applied to all groups to determine of expression of rEA from the Ad-GFP/rEA vector induced statistically higher levels of the factor tested. For ELISpot analysis, a one way ANOVA was used followed by Neuman-Keuls post hoc test (P<0.05). GraphPad Prism software was utilized for statistical analysis.

## Supporting Information

Methods S1 (0.03 MB DOC)

Figure S1 (A) C57BL/6 mice were injected with 1 ng rEA purified protein, 7.5×10^10^ vps Ad-GFP, a mixture of both rEA and Ad-GFP, or 7.5×10^10^ vp Ad-GFP/rEA. Plasma was harvested at 6 hpi and plasma cytokines and chemokines were analyzed. #, ## represent significantly different from mock injected animals using ANOVA and Dunnet's post hoc test (p<0.05, p<0.01 respectively). *,** represent statistically significant differences between data points using ANOVA and Newman-Keuls post hoc test (p<0.05, p<0.01, p<0.001, respectively). (B) MyD88-KO mice were injected with either Ad-GFP/rEA (n = 3) or UV inactivated Ad-GFP/rEA (n = 1) (7.5×10^10^ vp). At 1 and 6 h.p.i., plasma cytokines and chemokines were analyzed. * represents statistically significant inductions over mock treated mice (n = 3) using ANOVA and Dunnet's post hoc test (p<0.05). All bars represent mean ± SE.
(0.14 MB PDF)

Figure S2 (A) C57BL/6 or MyD88-KO mice were either mock injected or intravenously injected with 7.5×10^10^ vp of Ad-GFP, Ad-GFP/rEA, or UV treated Ad-GFP/rEA. Liver and spleen tissues were harvested at 6 hpi and analyzed for expression of the GFP transgene using Western blotting. Tubulin was probed as a loading control. This experiment was completed in triplicate with similar results. (B) RNA was also prepared from both liver and spleen tissues and analyzed for the presence of GFP and rEA transcript using semi-quantitative RT-PCR. GAPDH was used as a template control. All samples were evaluated in triplicate with equivalent results.
(0.16 MB PDF)

Figure S3 C57BL/6 mice were mock injected (n = 4), or intravenously injected with 7.5×10^10^ vp of Ad-GFP, Ad-GFP/rEA, or Ad-GFP/rEA pre-treated with 800 mws UV radiation (N = 5 per group). Plasma was harvested at 1, and 6 hpi. Cytokine induction was evaluated using Bio-Plex multiplex bead based ELISA. Bars represent mean ± SE. * denotes p<0.05, ** denotes p<0.01.
(0.11 MB PDF)

Figure S4 C57BL/6 mice (n = 4-5) were intravenously injected with 7.5×10^10^ vp of either Ad-GFP or Ad-GFP/rEA. (A) Blood platelets were enumerated at 1 and 3 dpi. Data points represent mean ± SD. ** represents a significant difference between Ad-GFP, and Ad-GFP/rEA treatment groups at the indicated time point (p<0.01) using one way ANOVA followed by a Neuman-Keuls post hoc test. # denotes a statistically significant decrease compared to the preceding time point (p<0.05) using a homoscedastic two tailed t-test. (B) Plasma was analyzed at time 0, as well as 1, 3, 7 and 28 d.p.i, for ALT activity. Data points represent mean ± SD.
(0.09 MB PDF)

Figure S5 C57BL/6 mice (N = 3) were either mock injected or injected with 7.5×10^10^ vps of either Ad-GFP or Ad-GFP/rEA. Lymphocytes from liver and spleen tissue were harvested at 6 hpi, stained for expression of surface markers for (A) NK cells or NKT cells and FACS sorted. Bars represent mean ± SE. *,**,*** represent statistical differences compared to mock (p<0.05, p<0.01, p<0.001, respectively). Representative contour plots are illustrated at right. Note: the gating strategy for NKT cells appears to miss significant portions of the NKT cell population in the spleen. To address this issue, we completed multiple analyses using various strategies in an attempt to include as much as the NKT cell population as possible. All strategies yielded equivalent results. (B) Splenic derived lymphocytes were also separated into the various indicated populations. Multiple gating strategies were also applied to the changing Cd11b+ population, each yielding equivalent results. *** represents a statistical difference from mock (p<0.001).
(0.74 MB PDF)

Figure S6 C57BL/6 mice were vaccinated IM with 1×10^7^ vps of Ad-Gag + the indicated doses of either PBS or purified rEA protein. Balb/c mice were vaccinated with 1×10^6^ vps +100 ng of purified rEA protein. (A) Anti Gag specific antibodies were titered from serum at various dilutions. (B) IFNγ or (C) IL-2 ELISpots were completed to quantify antigen specific T-cell responses.
(0.11 MB PDF)

Figure S7 HEK 293 cells were infected with the indicated viral dilutions. Each virus was normalized to 1×10^10^ viral particles/ml prior to dilutions in order to directly compare transduction efficiencies. At 24 hours post infection, % GFP positive cells were determined using FACS analysis on an LSRII flow cytometer.
(0.10 MB PDF)

Figure S8 Depletion resulted in >96% reduction of CD8+ cells. The viability of recovered CD8 depleted cells was >92% as measured by trypan blue viability staining.
(0.08 MB PDF)
