# Supplementary material for: A New Adenovirus Based Vaccine Vector Expressing an Eimeria tenella Derived TLR Agonist Improves Cellular Immune Responses to an Antigenic Target
Source: PLoS One. 2010 Mar 8;5(3):e9579. doi: 10.1371/journal.pone.0009579 (PMC2833191; doi:10.1371/journal.pone.0009579)
Supplement: Methods S1 — (0.03 MB DOC) [file pone.0009579.s001.doc]

**Supplemental Methods:**

**Western blotting and sqRT-PCR.** Liver and spleen tissue was homogenized in lysis buffer (20mM Tris-HCl, pH 7.4, 1mM EDTA, 150mM NaCl) containing 1% Triton X-100 with protease inhibitors, centrifuged at maximum speed (13,000 ×g) for 10 min at 4°C and quantified using the Bradford method. Equivalent concentrations (5 µg) of protein samples were run on polyacrylamide gels and transferred onto nitrocellulose membranes. Blots were probed with anti-GFP antibody (Santa Cruz, Santa Cruz, CA) followed by HRP conjugated secondary antibody (Sigma-Aldrich, St. Louis, MO). Semi-quantitative RT-PCR was carried out on total RNA harvested from spleen and liver tissue using primers specific for GFP or rEA. The cycle number used for amplification was optimized for each PCR reaction such that significant differences in gene expression could be visualized. Each experiment was conducted in triplicate with similar results.

**Plasma ALT levels as a measure of liver toxicity.** Evidence of liver toxicity was quantified by measuring plasma alanine aminotransferase (ALT) activity levels in plasma collected at 24hpi. ALT activity was determined spectrophotometrically using Infinity-ALT from Thermoelectron Corp per the manufacturer’s protocol (Louisville, CO).

**Platelet enumeration.** To assess Ad vector induced thrombocytopenia, platelets were measured at the respective time points by using the Unopette (Fisher-Scientific) system as previously described.[1,2] Per manufacture recommendations, RBCs were lysed, and platelets were counted using a Neubauer hemocytometer. Statistically significant differences for platelet measurements were determined using ANOVA with Dunnet’s post-hoc test, as compared to platelet counts determined in mock injected animals.

1. Hartman ZC, Kiang A, Everett RS, Serra D, Yang XY, et al. (2007) Adenovirus infection triggers a rapid, MyD88-regulated transcriptome response critical to acute-phase and adaptive immune responses in vivo. J Virol 81: 1796-1812.

2. Kiang A, Hartman ZC, Everett RS, Serra D, Jiang H, et al. (2006) Multiple innate inflammatory responses induced after systemic adenovirus vector delivery depend on a functional complement system. Mol Ther 14: 588-598.
