# Supplementary figures and images for: A New Adenovirus Based Vaccine Vector Expressing an Eimeria tenella Derived TLR Agonist Improves Cellular Immune Responses to an Antigenic Target
Source: PLoS One. 2010 Mar 8;5(3):e9579. doi: 10.1371/journal.pone.0009579 (PMC2833191; doi:10.1371/journal.pone.0009579)

**A**

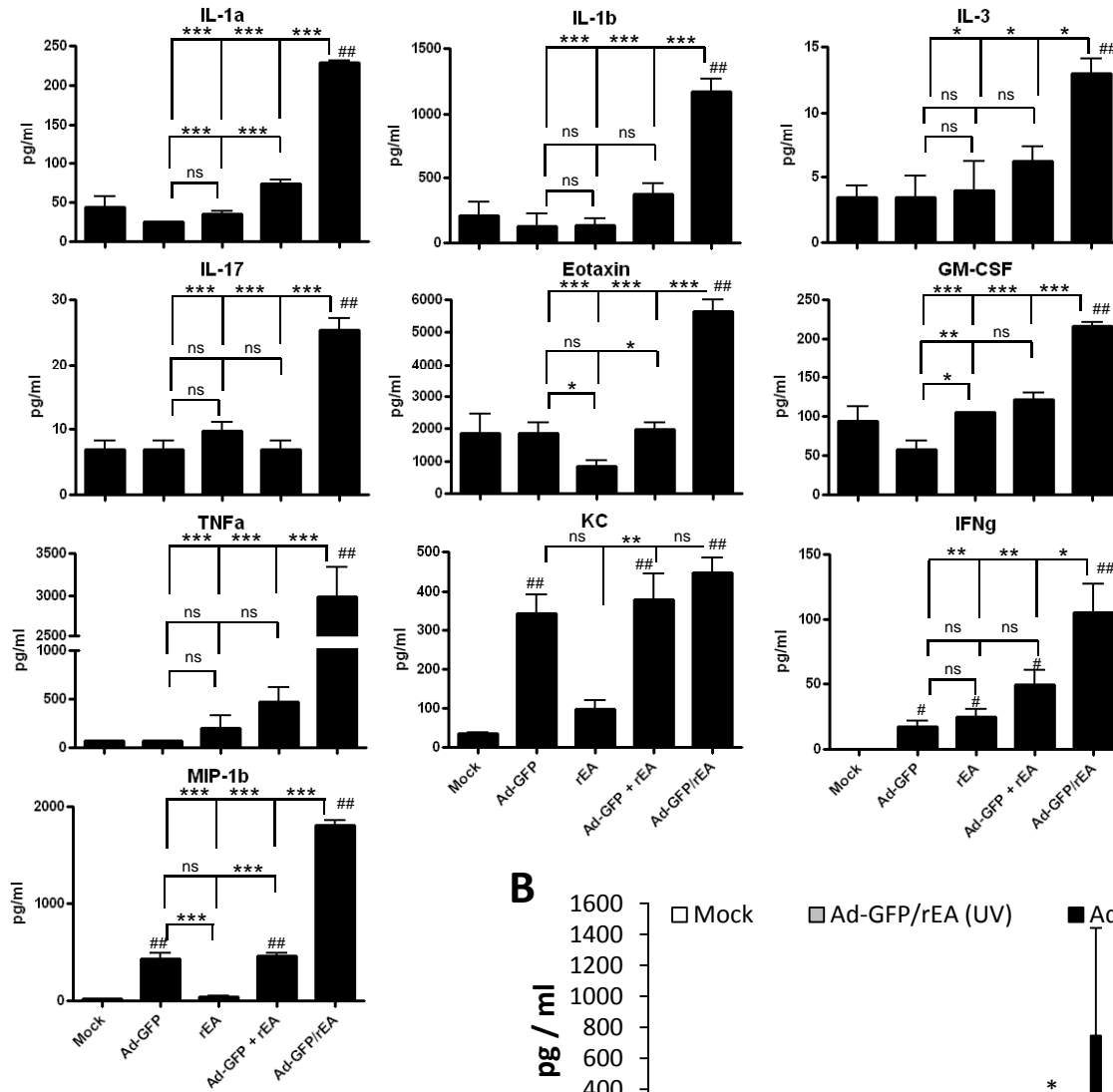

**B**

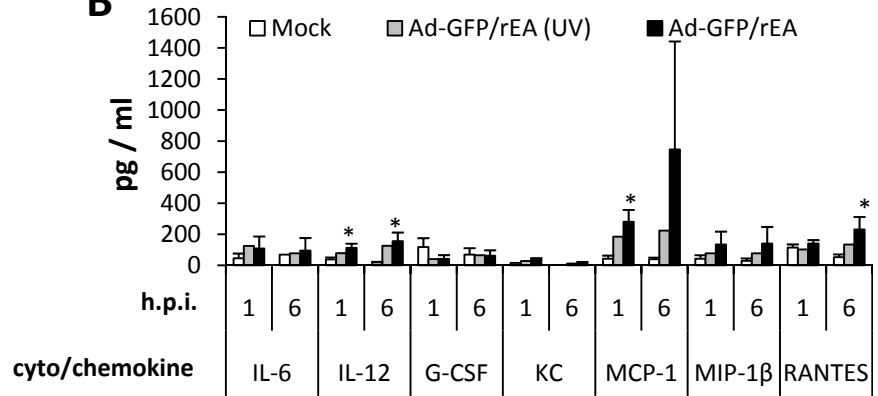

Supplement: Figure S1 — (A) C57BL/6 mice were injected with 1 ng rEA purified protein, 7.5×10^10^ vps Ad-GFP, a mixture of both rEA and Ad-GFP, or 7.5×10^10^ vp Ad-GFP/rEA. Plasma was harvested at 6 hpi and plasma cytokines and chemokines were analyzed. #, ## represent significantly different from mock injected animals using ANOVA and Dunnet's post hoc test (p<0.05, p<0.01 respectively). *,** represent statistically significant differences between data points using ANOVA and Newman-Keuls post hoc test (p<0.05, p<0.01, p<0.001, respectively). (B) MyD88-KO mice were injected with either Ad-GFP/rEA (n = 3) or UV inactivated Ad-GFP/rEA (n = 1) (7.5×10^10^ vp). At 1 and 6 h.p.i., plasma cytokines and chemokines were analyzed. * represents statistically significant inductions over mock treated mice (n = 3) using ANOVA and Dunnet's post hoc test (p<0.05). All bars represent mean ± SE. (0.14 MB PDF) [file pone.0009579.s002.pdf]

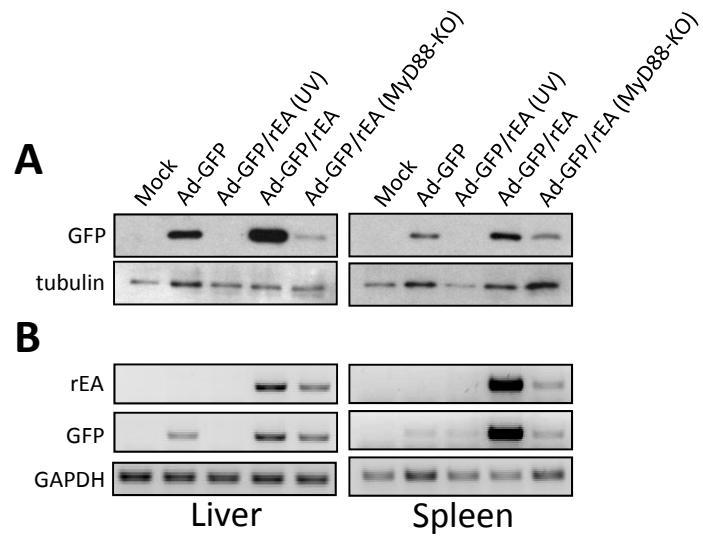

Supplement: Figure S2 — (A) C57BL/6 or MyD88-KO mice were either mock injected or intravenously injected with 7.5×10^10^ vp of Ad-GFP, Ad-GFP/rEA, or UV treated Ad-GFP/rEA. Liver and spleen tissues were harvested at 6 hpi and analyzed for expression of the GFP transgene using Western blotting. Tubulin was probed as a loading control. This experiment was completed in triplicate with similar results. (B) RNA was also prepared from both liver and spleen tissues and analyzed for the presence of GFP and rEA transcript using semi-quantitative RT-PCR. GAPDH was used as a template control. All samples were evaluated in triplicate with equivalent results. (0.16 MB PDF) [file pone.0009579.s003.pdf]

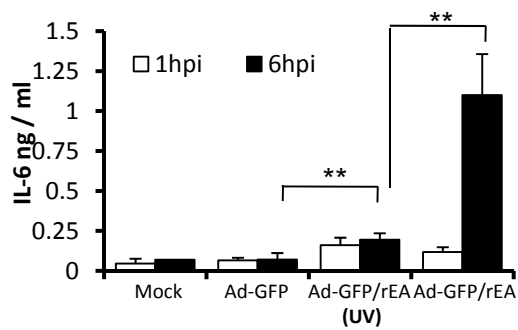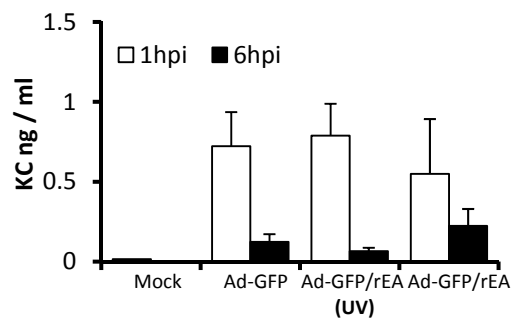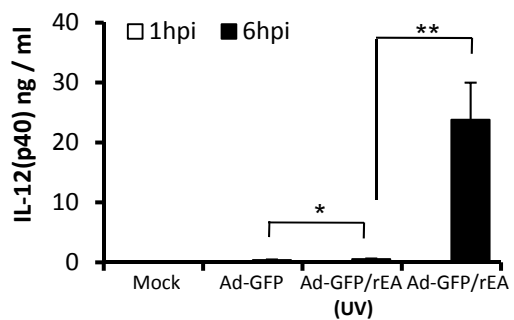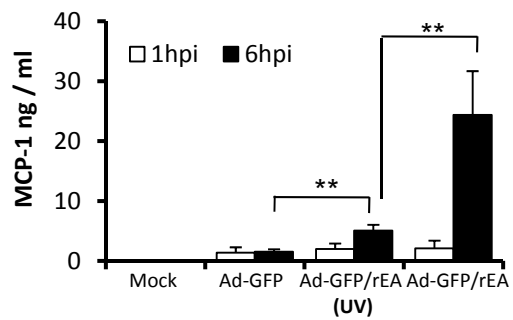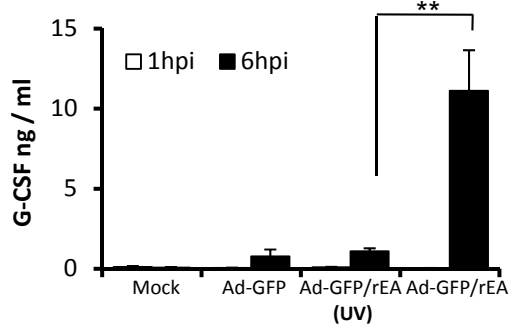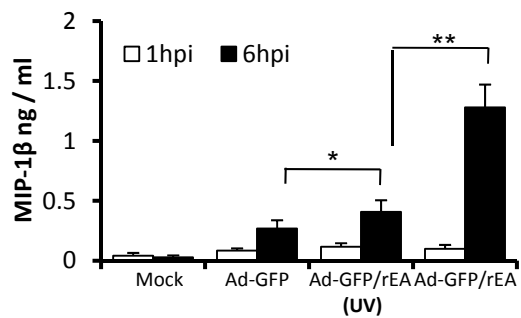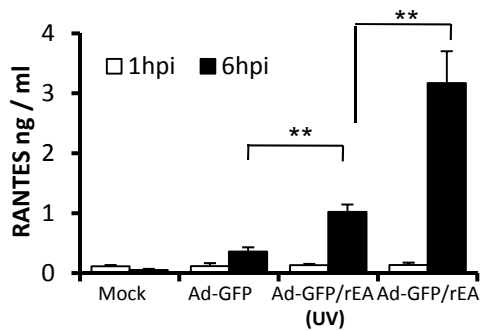

Supplement: Figure S3 — C57BL/6 mice were mock injected (n = 4), or intravenously injected with 7.5×10^10^ vp of Ad-GFP, Ad-GFP/rEA, or Ad-GFP/rEA pre-treated with 800 mws UV radiation (N = 5 per group). Plasma was harvested at 1, and 6 hpi. Cytokine induction was evaluated using Bio-Plex multiplex bead based ELISA. Bars represent mean ± SE. * denotes p<0.05, ** denotes p<0.01. (0.11 MB PDF) [file pone.0009579.s004.pdf]

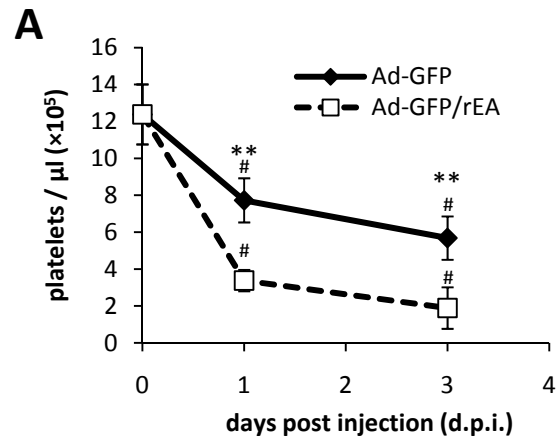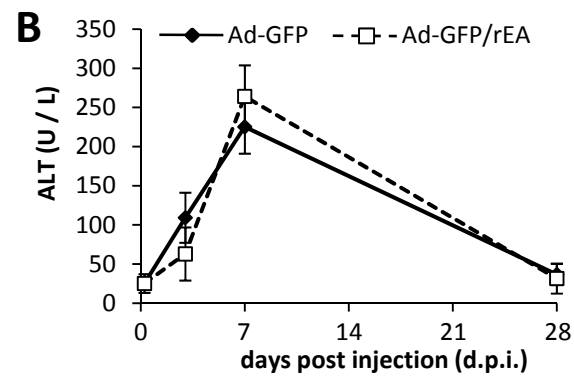

Supplement: Figure S4 — C57BL/6 mice (n = 4-5) were intravenously injected with 7.5×10^10^ vp of either Ad-GFP or Ad-GFP/rEA. (A) Blood platelets were enumerated at 1 and 3 dpi. Data points represent mean ± SD. ** represents a significant difference between Ad-GFP, and Ad-GFP/rEA treatment groups at the indicated time point (p<0.01) using one way ANOVA followed by a Neuman-Keuls post hoc test. # denotes a statistically significant decrease compared to the preceding time point (p<0.05) using a homoscedastic two tailed t-test. (B) Plasma was analyzed at time 0, as well as 1, 3, 7 and 28 d.p.i, for ALT activity. Data points represent mean ± SD. (0.09 MB PDF) [file pone.0009579.s005.pdf]

**A**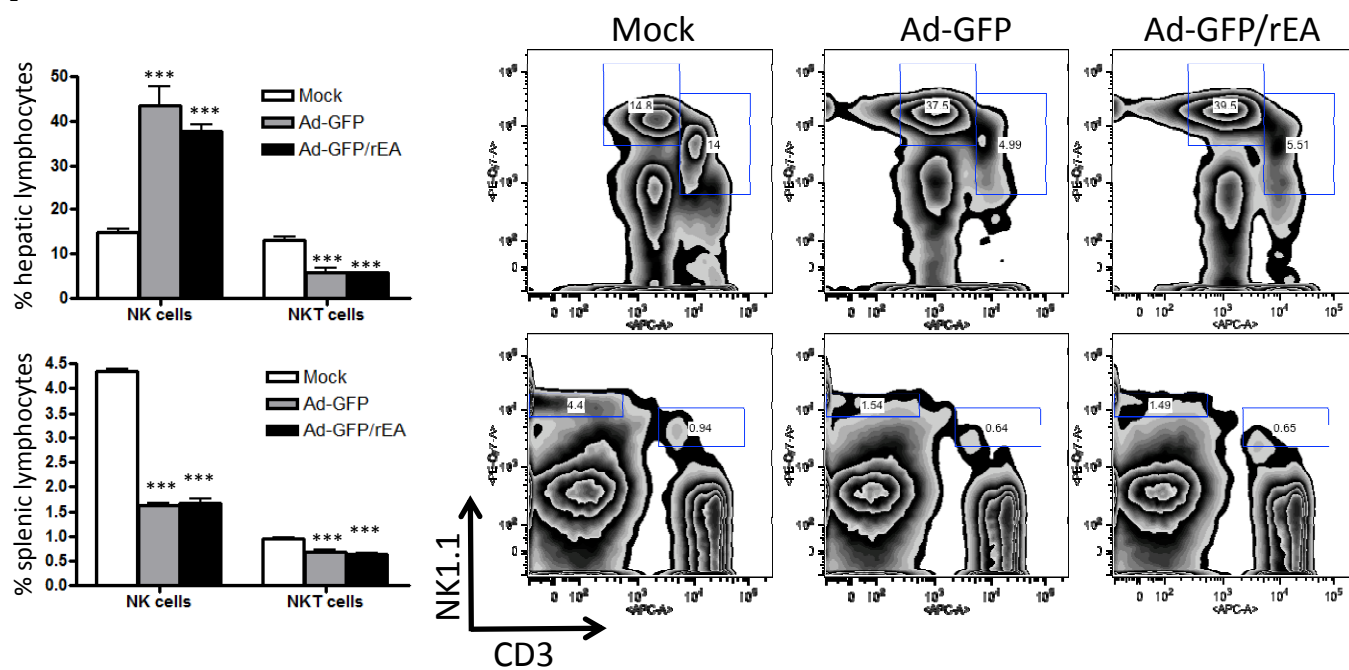**B**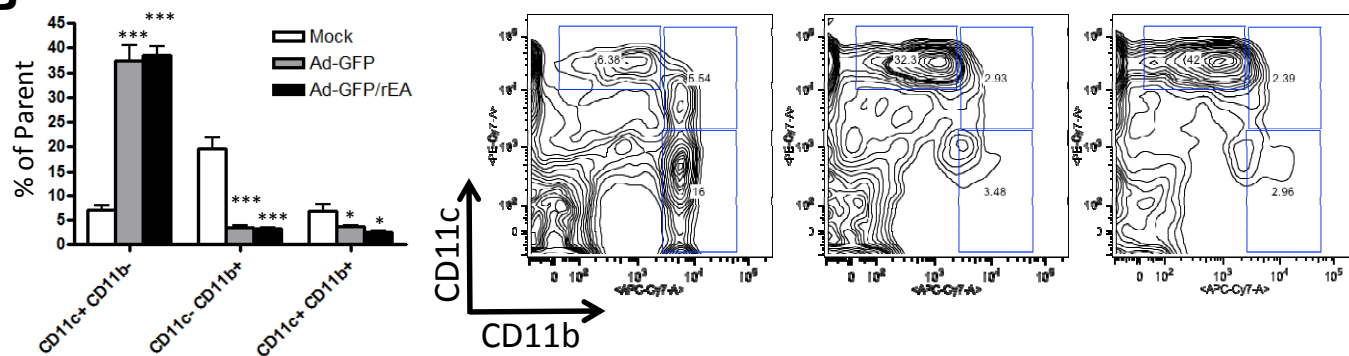

Supplement: Figure S5 — C57BL/6 mice (N = 3) were either mock injected or injected with 7.5×10^10^ vps of either Ad-GFP or Ad-GFP/rEA. Lymphocytes from liver and spleen tissue were harvested at 6 hpi, stained for expression of surface markers for (A) NK cells or NKT cells and FACS sorted. Bars represent mean ± SE. *,**,*** represent statistical differences compared to mock (p<0.05, p<0.01, p<0.001, respectively). Representative contour plots are illustrated at right. Note: the gating strategy for NKT cells appears to miss significant portions of the NKT cell population in the spleen. To address this issue, we completed multiple analyses using various strategies in an attempt to include as much as the NKT cell population as possible. All strategies yielded equivalent results. (B) Splenic derived lymphocytes were also separated into the various indicated populations. Multiple gating strategies were also applied to the changing Cd11b+ population, each yielding equivalent results. *** represents a statistical difference from mock (p<0.001). (0.74 MB PDF) [file pone.0009579.s006.pdf]

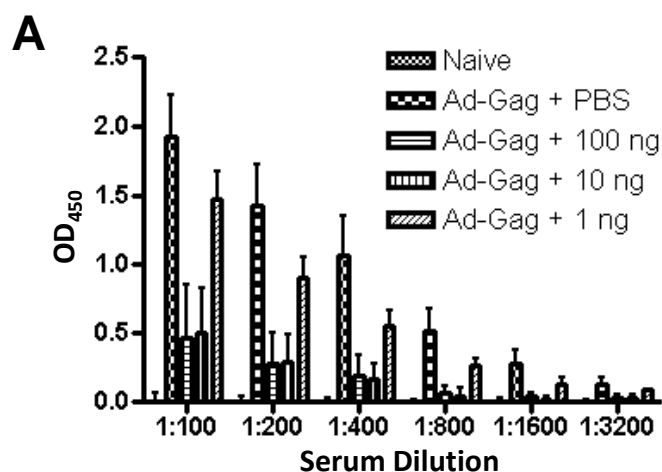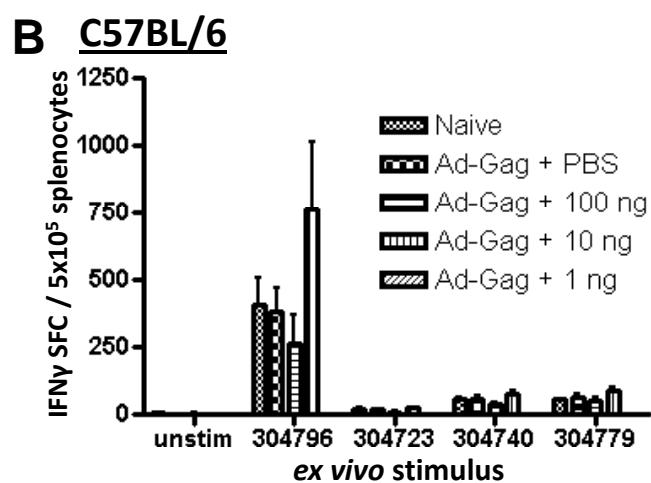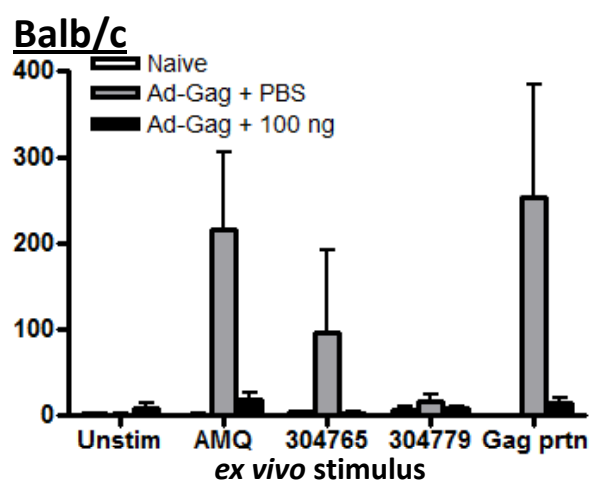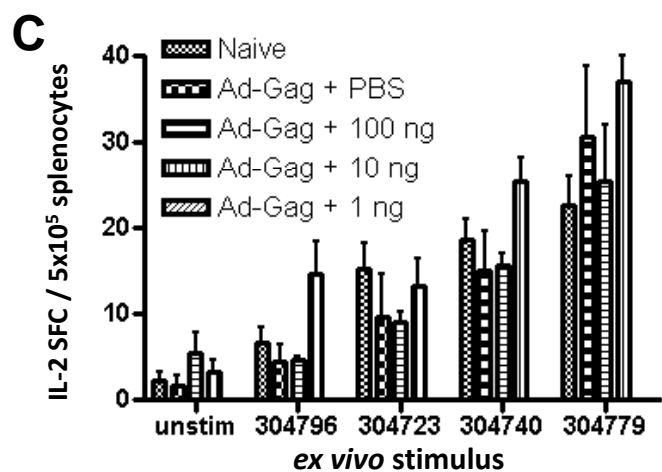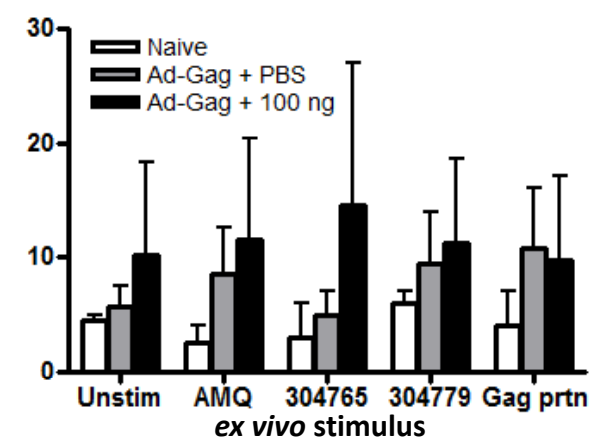

Supplement: Figure S6 — C57BL/6 mice were vaccinated IM with 1×10^7^ vps of Ad-Gag + the indicated doses of either PBS or purified rEA protein. Balb/c mice were vaccinated with 1×10^6^ vps +100 ng of purified rEA protein. (A) Anti Gag specific antibodies were titered from serum at various dilutions. (B) IFNγ or (C) IL-2 ELISpots were completed to quantify antigen specific T-cell responses. (0.11 MB PDF) [file pone.0009579.s007.pdf]

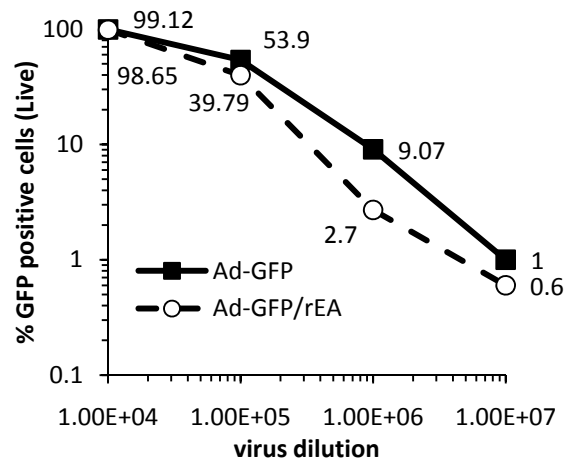

Supplement: Figure S7 — HEK 293 cells were infected with the indicated viral dilutions. Each virus was normalized to 1×10^10^ viral particles/ml prior to dilutions in order to directly compare transduction efficiencies. At 24 hours post infection, % GFP positive cells were determined using FACS analysis on an LSRII flow cytometer. (0.10 MB PDF) [file pone.0009579.s008.pdf]

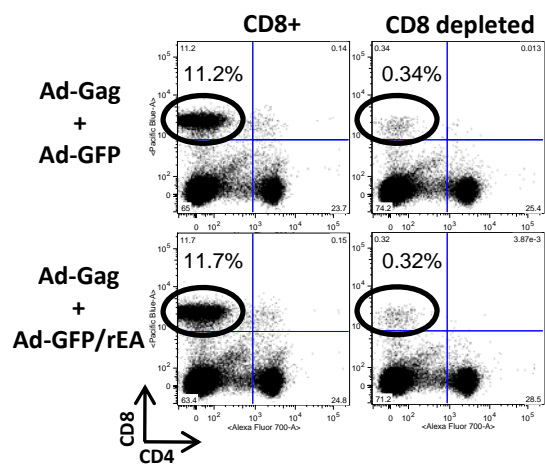

Supplement: Figure S8 — Depletion resulted in >96% reduction of CD8+ cells. The viability of recovered CD8 depleted cells was >92% as measured by trypan blue viability staining. (0.08 MB PDF) [file pone.0009579.s009.pdf]
